# The Amended Syllabus of Nursing Education

**Published:** 1921-09-24

**Authors:** 


### THE AMENDED SYLLABUS OF NURSING EDUCATION.

Although the Draft Syllabus put forward some months ago by the Education Committee of the General Nursing Council met with a very cordial reception from the profession generally, there were not wanting critics who complained of it as too comprehensive and unsuited as a curriculum for the rank and file of nurses. Full opportunity has been granted for discussion and objections have received careful consideration. A conference was held to which representatives of the institutions affected by the Syllabus were invited and great care has been taken to remove any misapprehension and repair omissions. The Syllabus as re-issued, no longer in draft form, presents nevertheless very few alterations, and we may take this as a mark of its general acceptance by those concerned to carry it out.

We note first with much satisfaction the insertion of the words "District Nursing" among the subjects, Public Health and Sanitation, of growing importance in the training of a nurse, which should be brought to her notice from the outset and kept in full view throughout her training.

In the first year's training, "rectal and vaginal examinations" are qualified by the words "preparation for," the former condensed expression having led to a misunderstanding as to the scope of the knowledge to be imparted. There are no omissions in this part of the Syllabus, which is significant, and one useful addition under food values, viz., "proprietary preparations for infants: their value and dangers."

In the second and third year's course we note the addition of "anæsthesia, general, spinal-local," also the introduction of "massage exercises and medical electricity, their general principles and therapeutic value," in connection with diseases of bones and joints. "Magnetism and electricity" are introduced into elementary science subjects, and "diseases of the eye" follow on after those of the nose, ear, and throat." The nurses' chart remains untouched.

The result cannot fail to be very gratifying to the leaders in nursing education who are responsible for the Syllabus. It may fairly be claimed that no educational programme has ever been put forth which affects so vitally the future alike of voluntary and municipal institutions. It has been subjected to a searching fire of objections, chief among which was the ignorant cry that it was overladen with matter. It emerges without the loss of a single item and with the addition of some half-dozen new subjects. It remains to be shown in what manner it may most advantageously be put into use in large and small training-schools.

